# Association between bone mineral density and lower back pain in the general United States population using the NHANES of 1999–2004

**DOI:** 10.3389/fsurg.2025.1535614

**Published:** 2025-04-10

**Authors:** Yang Zhou, Chi Li, WangYing Dai, HongLin Teng, Yu Wang, MingYu Zhu, Jing Wang

**Affiliations:** Department of Spinal Surgery, The First Affiliated Hospital of Wenzhou Medical University, Wenzhou, China

**Keywords:** lower back pain, bone mineral density, osteoporosis, economic impact, U-shaped relationship

## Abstract

**Objectives:**

Lower back pain (LBP) is a prevalent health issue that has substantial effects on individuals and society. However, the association between bone mineral density (BMD) and LBP remains controversial. In this study we aimed to ascertain whether a relationship exists between BMD and LBP in the United States population.

**Methods:**

Data from the National Health and Nutrition Examination Survey (NHANES) spanning 1999–2004 were analyzed using a cross-sectional approach. BMD and LBP were assessed using multivariate logistic regression, smoothing curves, and generalized additive models. Subgroup analyses were also performed to ensure data stability and mitigate confounding factors.

**Results:**

In this population-based study, the data of 107,570 adults were analyzed (mean age: 47.13 ± 18.38 years) and 36.74% of them had LBP. After controlling for all covariates, a positive correlation was established between BMD and LBP [odds ratio (OR) = 1.87, 95% confidence interval (CI) = (1.00, 3.50)]. The two-segment linear regression model revealed a U-shaped relationship between BMD and LBP with a 1.14 g/cm^2^ inflection point. BMD values <1.14 g/cm^2^ were linked to a lower likelihood of experiencing LBP [OR = 0.55, 95% CI = (0.45, 0.68)]. However, a BMD >1.14 g/cm^2^ increased the risk of LBP [OR = 6.15, 95% CI = (4.51, 8.39)].

**Conclusions:**

BMP was significantly and positively correlated with LBP. A U-shaped relationship was observed between BMD and LBP, indicating that both insufficient and excessive BMD may increase the risk of LBP.

## 1 Introduction

Defined as discomfort in the lower back, lumbosacral, and sacroiliac areas, lower back pain (LBP) frequently includes radiating pain in the lower limbs (1). It is a prevalent condition affecting approximately 84% of individuals during their lifetime (2). Globally, LBP poses substantial social and economic difficulties and causes extensive workplace absenteeism (3). LBP is estimated to incur annual costs of $34 billion in the United States alone, with additional indirect expenses, such as lost wages, potentially exceeding $100 billion (4). Given its widespread occurrence and substantial economic impact on healthcare systems, the implementation of early screening measures for LBP in populations at high risk using known risk factors, alongside the development of effective treatment protocols, is crucial.

Osteoporosis manifests as diminished bone mass and structural deterioration, leading to reduced skeletal stability and a higher probability of fracture (5). This condition in adults is defined as a bone mineral density (BMD) that is 2.5 standard deviations below their maximum bone density (6). Osteoporotic fractures, the primary complications of this disease, frequently occur in older adults and are associated with a significantly high mortality risk from major fractures (7, 8).

LBP is categorized into non-specific and specific types, depending on its origin. Prior research has pinpointed risk factors for nonspecific LBP, including sex, age, educational level, depression, seated posture, working hours, accidents, and genetic factors (9–11). Additionally, specific types of LBP may result from structural changes in the spine such as spinal stenosis, arthritis, disc degeneration, and kyphoscoliosis (12, 13). Despite numerous studies highlighting the risk factors for LBP, the role of osteoporosis as a contributing factor has often been ignored; however, it may be crucial.

Gaber et al. reported that small sample sizes showed a correlation between LBP and reduced bone density, indicating a potential risk of osteopenia (14). However, individuals with LBP may have higher lumbar BMD than those without, indicating a potential connection between issues such as rotational asymmetry or limited motion and increased bone density in the affected vertebrae (15). However, no consensus has been reached in the academic literature regarding the potential association between osteoporosis and LBP.

This study investigated the association between osteoporosis and LBP, using an extensive cross-sectional study derived from the National Health and Nutrition Examination Survey (NHANES) dataset.

## 2 Materials and methods

### 2.1 Study population

**Figure 1:**
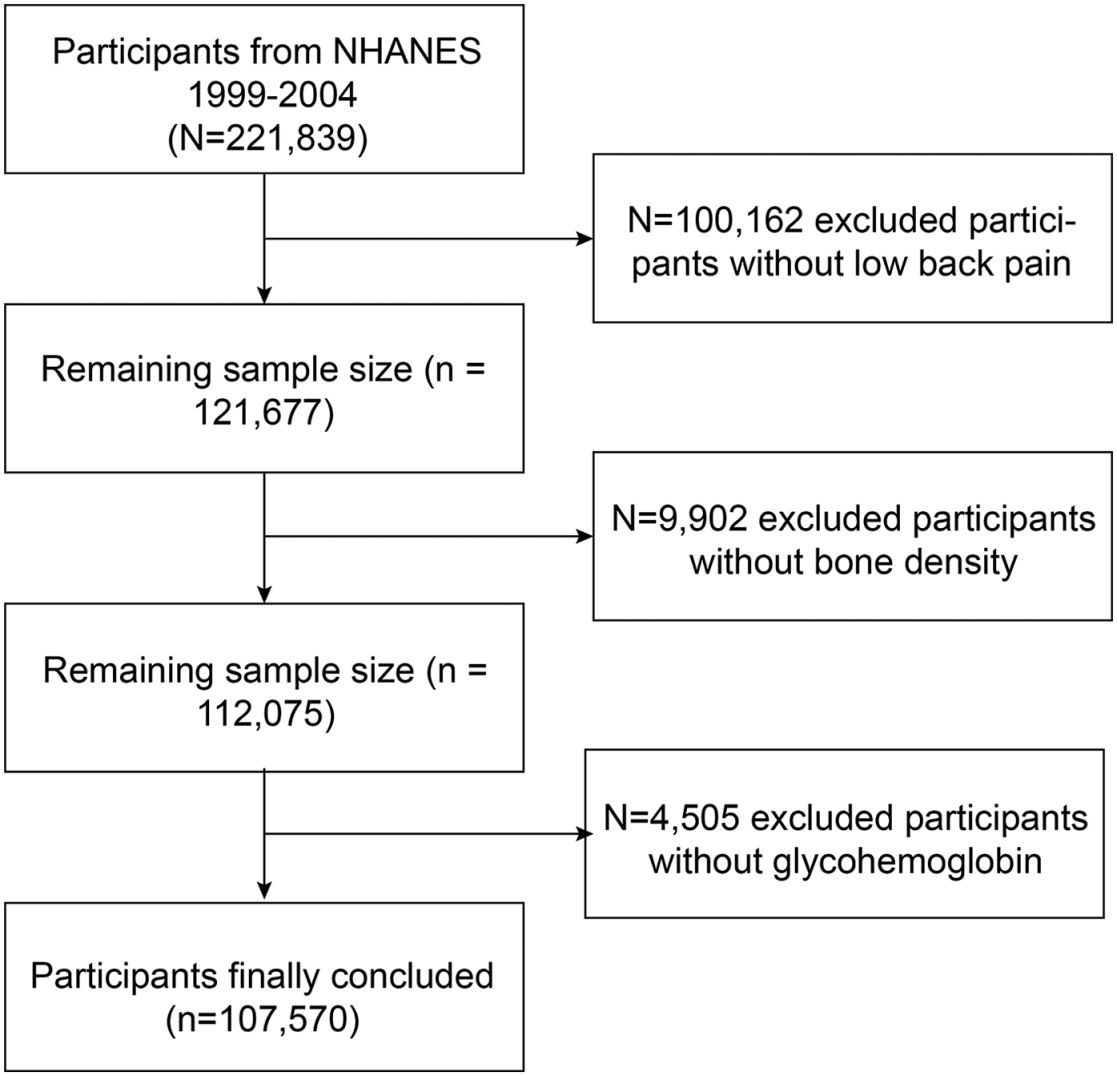
Flowchart of the participants selection from NHANES 1999–2004.

Owing to the limited data availability on LBP in recent years, supplementary data were collected from the NHANES iterations during 1999–2000, 2001–2002, and 2003–2004. A total of 221,839 individuals completed questionnaires on nutrition and health conditions and underwent health examinations. Only 107,570 participants were selected for inclusion in the study because of a lack of data on LBP (*n* = 100,162), BMD (*n* = 9,602), and glycohemoglobin levels (*n* = 4,505) (Figure 1). The database is now publicly accessible and authorized for researcher use, contingent on the acquisition of informed consent from all participants involved in the studies.

**Figure 2:**
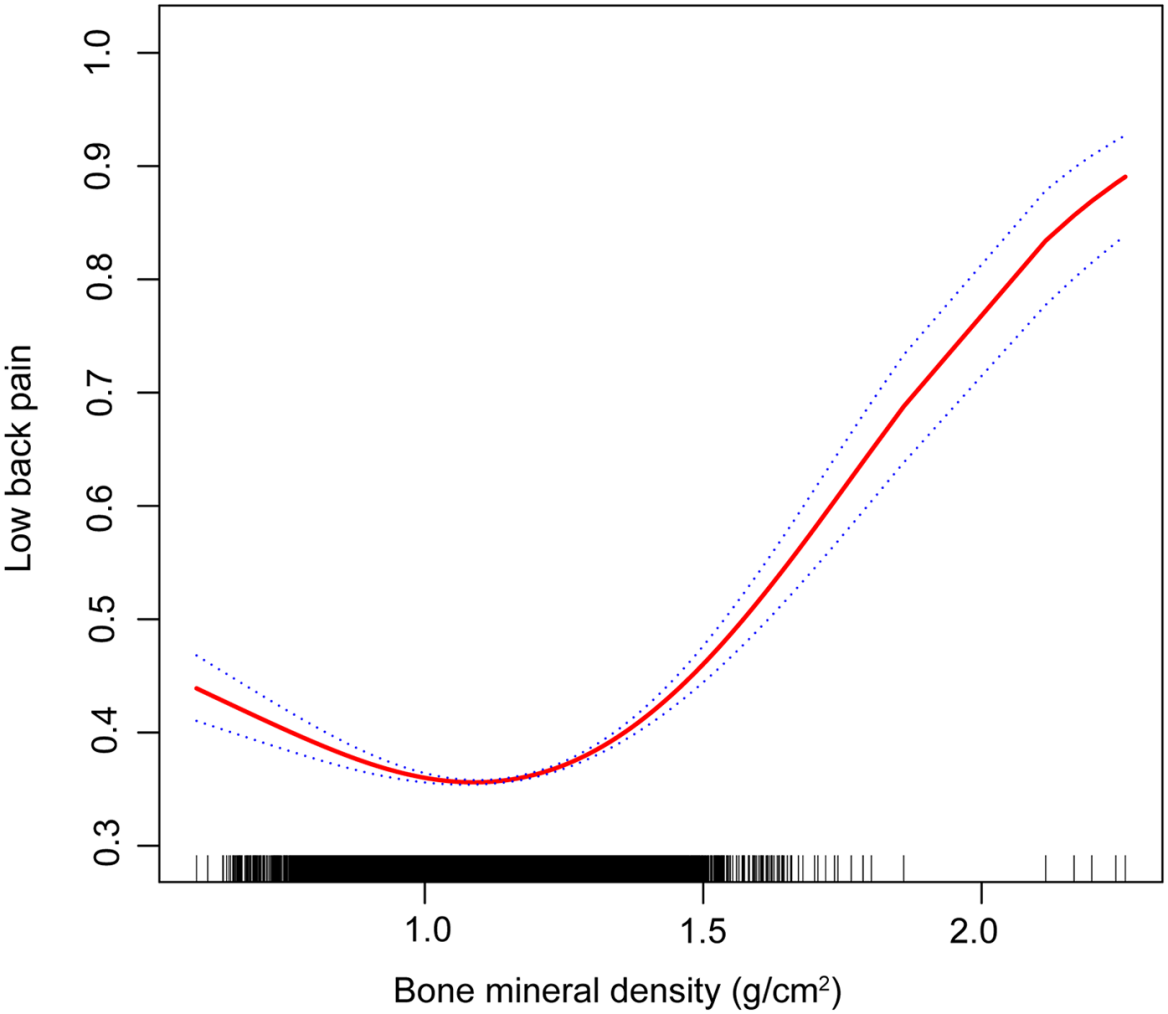
Association between bone mineral density and lower back pain (the solid red line represents the smooth curve fit between variables. Blue bands represent the 95% confidence interval from the fit).

### 2.2 Bone mineral density

The final analysis included dual-energy x-ray absorptiometry (DXA) examinations using Hologic QDR-4500A densitometers (Hologic, Marlborough, MA, USA) conducted by certified radiology technologists for all participants. Data were analyzed using Hologic APEX software (Hologic, Marlborough, MA, USA). Further information can be found on the NHANES website.

### 2.3 Lower back pain

Data on LBP was collected from participants aged ≥20 years who filled out pain questionnaires. Questions about LBP were answered based on the past 3 months. Furthermore, the pain was supposed to last a full day or longer and not just be mild or temporary. The specific survey questions were as follows: “During the past 3 months, did {you/SP} have LBP?” All the aforementioned data were processed as “not available” data because participants declined to respond, replied, “I do not know”, or were absent.

### 2.4 Other covariates

The covariates in our analysis were mostly classified into 3 categories: (1) Demographics: age, sex, racial/ethnic background (Mexican American, Other Hispanic, Non-Hispanic, Non-Hispanic Black people, Other race), family poverty-to-income ratio (PIR) (<1, 1–3, >3), and educational attainment (below high school, high school, above high school); (2) Medical conditions: Body mass index (BMI) (underweight if <18.5; normal weight if ≥18.5 and ≤24.9; overweight if ≥25 and ≤29.9; obese if ≥30), hyperlipidemia (whether or not the individual had been informed that their blood cholesterol level was high), glycohemoglobin; (3) Lifestyle variables included moderate physical activity over the past 30 days, alcohol consumption (individuals reporting the consumption of at least 12 alcoholic beverages per year), and smoking status (individuals reporting smoking at least 100 cigarettes over their lifetime).

### 2.5 Statistical analysis

Weights were produced for complicated sample designs using Mobile Examination Center (MEC) exam sampling weights, which were used to weigh all the data before analysis. Data with a weighting of zero were removed. Two-year sample MEC exam weights (WTMEC2YR) were used for data from the 2003–2004 cycle and four-year sample MEC exam weights (WTMEC4YR) were used for data from the 1999–2002 cycle. The weights were calculated using SDMVPSU and SDMVSTRA variables.

Categorical variables were presented as percentages, whereas continuous variables were described as means and standard deviations. The linear association between BMD and LBP was investigated using weighted multiple linear regression. Curve fitting and threshold effect analyses were used to determine the non-linear relationship between these variables. This research employed three distinct models: Model 1, without adjustment; Model 2, which included adjustments for age, sex, and race; and Model 3, which adjusted for all covariates, as detailed in Table 1. Additional subgroup analyses were also performed. To explore the non-linear dynamics between BMD and LBP, both a generalized additive model and a smoothing curve were applied. Upon detecting non-linearity, the inflection point was identified using a recursive algorithm, followed by the construction of separate binary linear regression models on either side of the point. The log-likelihood ratio test was used to evaluate the statistical significance of segmented logistic regression models. All analyses were performed using R software (version 4.0.3, available at https://www.R-project.org) and Empower Stats (version 6.0, available at https://www.empowerstats.com), with the significance threshold set at *P* < 0.05.

**Table 1:** Association between BMD and LBP.

Model	Low back pain (95% CI)	Adjusted analysis *P*-value
Model 1	0.97 (0.62, 1.54)	0.9243
Model 2	1.39 (0.84, 2.41)	0.2439
Model 3	1.87 (1.00, 3.50)	0.0470

Model 1: no covariates were adjusted.

Model 2: age, gender, and race were adjusted.

Model 3: age, sex, race, education level, PIR, BMI, smoking habit, alcohol habit, smoke, hyperlipidemia, glycohemoglobin and physical activity were adjusted.

## 3 Results

### 3.1 Population characteristics

Table 2 presents the demographic profiles of the study participants. Among the adults surveyed, 36.74% reported experiencing LBP. These individuals had an average age of 47.13 ± 18.38 years, with men constituting 52.89% of the group. Typically, individuals with LBP tend to be women, predominantly non-Hispanic White people, with less education and lower PIRs. They also generally had higher BMIs, were nonsmokers, lacked hyperlipidemia, and engaged in moderate physical activity. Additionally, the age, drinking habits, and glycohemoglobin levels were similar between the two groups.

**Table 2:** Characteristics of the participants.

Characteristic	Low back pain		*P*-value	*P*-interaction
	NO, *n* = 68,045 (63.26%)	Yes, *n* = 39,525 (36.74%)		
Gender				
Male	53.54 (52.14, 54.93)	49.69 (47.61, 51.77)	<0.01	0.44
Female	46.46 (45.07, 47.86)	50.31 (48.23, 52.39)		
Age (years)				
<60	83.12 (81.83, 84.33)	83.54 (81.80, 85.14)	0.65	0.11
>60	16.88 (15.67, 18.17)	16.46 (14.86, 18.20)		
Race (%)				
Mexican American	6.01 (4.72, 7.63)	5.37 (4.11, 6.99)	0.04	0.39
Other Hispanic	5.00 (3.51, 7.07)	5.83 (3.90, 8.62)		
Non-Hispanic White	74.84 (71.46, 77.95)	76.76 (73.49, 79.74)		
Non-Hispanic Black	9.21 (7.61, 11.11)	8.32 (6.63, 10.39)		
Other race	4.93 (3.90, 6.22)	3.72 (2.73, 5.05)		
Education (%)				
Less than high school	13.03 (11.73, 14.44)	17.02 (15.13, 19.09)	<0.01	0.42
High school	21.28 (19.50, 23.17)	26.24 (23.53, 29.14)		
More than high school	65.57 (63.19, 67.86)	56.70 (53.16, 60.17)		
Not recorded	0.13 (0.05, 0.33)	0.05 (0.02, 0.11)		
PIR (%)				
<1.3	14.02 (11.90, 16.45)	17.97 (15.46, 20.78)	<0.01	0.72
1.3–3.5	29.47 (26.90, 32.17)	33.37 (31.08, 35.75)		
>3.5	49.75 (46.20, 53.31)	42.21 (39.05, 45.43)		
Not recorded	6.76 (5.49, 8.30)	6.46 (5.29, 7.86)		
BMI (kg/m^2^)				
<18.5	1.73 (1.33, 2.26)	1.72 (1.28, 2.30)	0.04	0.18
18.5–25	36.89 (34.71, 39.13)	33.53 (30.70, 36.49)		
25–30	35.48 (33.63, 37.38)	35.79 (33.20, 38.47)		
>30	25.21 (23.26, 27.28)	28.37 (26.09, 30.75)		
Smoke (%)				
Yes	56.47 (53.89, 59.01)	47.76 (43.78, 51.76)	<0.01	0.35
No	43.49 (40.95, 46.07)	52.19 (48.21, 56.15)		
Alcohol (%)				
Yes	22.40 (18.46, 26.90)	22.59 (18.46, 27.34)	0.97	0.85
No	72.46 (68.09, 76.45)	72.41 (67.87, 76.53)		
Not recorded	5.14 (4.22, 6.24)	5.00 (3.72, 6.68)		
Moderate PA in past 30 days				
Yes	30.34 (27.97, 32.83)	34.15 (31.02, 37.41)	<0.01	0.05
No	69.65 (67.16, 72.02)	65.82 (62.56, 68.93)		
Hyperlipidemia (%)				
Yes	46.47 (43.79, 49.17)	42.69 (40.55, 44.85)	<0.01	0.59
No	22.19 (20.39, 24.09)	26.80 (24.66, 29.07)		
Not recorded	31.34 (28.25, 34.61)	30.51 (27.78, 33.38)		
Glycohemoglobin	5.36 (5.32, 5.40)	5.39 (5.35, 5.44)	0.12	

Abbreviations: PIR, poverty-to-income ratio; BMI, body mass index, BMD, body mineral density; PA, physical activity.

**Table 3:** Threshold effect analysis of BMD on LBP.

Low back pain	Adjusted OR (95% CI)	*P*-value
Fitting by the standard linear model	1.46 (1.30, 1.65)	<0.01
Fitting by the two-piecewise linear model		
Inflection point	1.14 g/cm^2^	
BMD < 1.14 g/cm^2^	0.55 (0.45, 0.68)	<0.01
BMD ≥ 1.14 g/cm^2^	3.41 (2.83, 4.12)	<0.01
P for Log-likelihood ratio		<0.01

Adjusted for age, sex, race, education level, PIR, BMI, smoking habit, alcohol habit, smoke, hyperlipidemia, glycohemoglobin and physical activity.

### 3.2 Association between BMD and LBP

The results of the multivariate regression analysis are presented in Table 1 and Figure 2. No significant relationship was observed between BMD and LBP in Models 1 and 2. After adjustment for all covariates, BMD was positively associated with LBP [1.8769 (1.005, 3.5024)]. As shown by smoothed curve fitting, BMD was associated with LBP with a U-shaped curve (*P* for Log-likelihood ratio <0.001, Table 3). A significant decline was observed in LBP risk with increasing BMD. The lowest incidence of LBP occurred at a BMD of 1.14 g/cm^2^, after which the trend in the curve was reversed. In the subgroup analyses, no significant interactions were noted among sex, age, race, education, hyperlipidemia, BMI, smoking, drinking, PIR, and physical activity.

## 4 Discussion

This study revealed that BMD was positively associated with LBP using data from the NHANES from between 1999 and 2004. In our cross-sectional study involving 107,570 participants, we observed a U-shaped relationship between BMD and LBP, with an inflection point at 1.14 g/cm^2^. When BMD was <1.14 g/cm^2^, a negative association was observed between elevated BMD and LBP. However, once the BMD levels surpassed 1.14 g/cm^2^, a positive association was detected between BMD and LBP.

A population-based cross-sectional study revealed that independent of factors such as age, education, and medical history, higher lumbar spine bone density correlated with increased LBP (16). Research involving Taiwanese adults found that LBP frequently accompanies osteoporosis, with elevated risks among women, those with lower educational levels, and individuals in blue-collar occupations (17). Manabe et al. noted a significant link between higher BMD and LBP among middle-aged women (18). Evidence suggests that patients with osteoporosis can experience back pain without fractures; however, treatments such as monthly ibandronate (19) and alendronate sodium (20) can alleviate pain. Nevertheless, the studies mentioned above have certain limitations. The sample sizes used in these investigations were not sufficiently large to ensure robust representativeness. Additionally, the studies did not account for a range of potential confounding variables such as age, sex, ethnicity, educational attainment, PIR, smoking habits, BMI, and BMD. These oversights might have undermined the reliability of the study outcomes.

LBP induced by osteoporosis is a complex, multifactorial issue.

Osteoporosis-induced LBP is associated with biomechanical alterations in the spine. Pan et al. determined that higher bone density in the lumbar facet joints may suggest increased stress on the joints and an uneven distribution of weight, possibly contributing to LBP (21). Andersen et al. found that patients with degenerative spondylolisthesis had lower spinal bone density than those with spinal stenosis, suggesting a possible connection between low BMD and the onset of degenerative spondylolisthesis (22).

An imbalance in bone metabolism is another possible cause of LBP in patients with osteoporosis. In addition to increasing bone resorption, reduced bone formation also increases fragility, making the spine more susceptible to stress and damage (23). Drugs, such as neridronate and alendronate, can alleviate LBP by improving bone density and reducing the bone turnover rate.

Disc herniation frequently emerges as the primary cause of back pain in patients with osteoporosis. This study corroborated previous findings linking higher BMD *T*-scores with increased spinal sclerosis in individuals experiencing LBP (24). Slowly progressive degenerative changes in the lower back impair the spine structure and functionality. Mechanical stress from spinal motion stimulates disc and facet joints, potentially leading to osteophyte development and endplate sclerosis. Additionally, microfractures contribute to a localized surge in bone turnover, enhancing trabecular density and reducing trabecular spacing (23, 26). These changes lead to increased bone density within the vertebral bodies, often marked by new osteophytes and endplate sclerosis (27). Recent findings indicate that higher bone density in the vertebral bodies correlates with intensified degeneration of the adjacent intervertebral discs. Research using micro-computed tomography has established a positive relationship between vertebral bone density and the severity of disc degeneration, which supports our conclusions (28). Consequently, while stiff vertebrae may subject neighboring discs to greater mechanical stress, osteoporotic vertebrae may offer some degree of protection against such degeneration (18).

During lumbar degeneration, the load distribution on the spinal functional unit is altered, resulting in greater pressure being placed on the intervertebral discs causing microfractures in the endplate. The nucleus pulposus material in the intervertebral disc penetrates these microfractures into the endplate, triggering local inflammatory reactions and ultimately leading to Modic changes (29). Significant differences exist in trabecular bone microstructure and bone remodeling indices among the different types of Modic changes. Modic type 1 changes exhibit higher bone turnover, likely due to inflammatory processes, whereas Modic type 2 changes are associated with reduced bone formation (30). Modic changes are associated with LBP. A cross-sectional study revealed that Modic type 1 changes are significantly associated with chronic LBP, with a higher frequency and severity of pain. In contrast, the relationship between Modic type 2 and 3 changes and the LBP is weaker (31). Other studies indicate that Modic type 1 changes are critical factors in patients with LBP, correlating with pain, functional deterioration, and unsuccessful return to work within 1 year. Among the 325 patients on sick leave owing to LBP, those with Modic type 1 changes reported more severe back pain and no improvement in pain or disability (32). We believe that Modic changes may be a pathway by which bone density influences LBP.

Our findings demonstrate a significant link between BMD and the occurrence of LBP. The data suggest that lower BMD is commonly associated with an increased incidence of LBP, which is consistent with prior studies that connect osteoporosis with such pain. In contrast, a higher BMD appears to increase the risk of developing LBP, likely due to degenerative alterations in the intervertebral discs and articular joints. Clinicians should consider incorporating measures of BMD into routine assessments for patients with or at risk of LBP. Future research should explore the underlying mechanisms linking BMD and LBP in more detail and evaluate the effectiveness of targeted interventions aimed at preventing and managing LBP. This could lead to the development of more effective, personalized treatment strategies that improve patient outcomes and reduce the burden of LBP on individuals and healthcare systems.

Our study had several limitations. First, the cross-sectional design prevented the establishment of causal links between bone BMD and LBP. Second, although numerous covariates were accounted for in the multivariate regression analysis, the possibility of residual confounding factors remained.

## 5 Conclusions

The results of this study revealed a non-linear relationship between BMD and the prevalence of LBP among adults in the United States, showing a notable threshold effect. The risk of LBP increases when BMD levels are either too low or too high. However, a deeper understanding of this relationship will significantly aid future research efforts.

## Data Availability

The datasets presented in this study can be found in online repositories. The names of the repository/repositories and accession number(s) can be found below: https://www.cdc.gov/nchs/nhanes/.

## Supplementary material

Supplementary Image S1 The association between BMD and LBP, stratified by age.

Supplementary Image S2 The association between BMD and LBP, stratified by BMI.

Supplementary Image S3 The association between BMD and LBP, stratified by alcohol.

Supplementary Image S4 The association between BMD and LBP, stratified by education.

Supplementary Image S5 The association between BMD and LBP, stratified by Hyperlipidemia.

Supplementary Image S6 The association between BMD and LBP, stratified by Hyperlipidemia.

Supplementary Image S7 The association between BMD and LBP, stratified by moderate physical activity.

Supplementary Image S8 The association between BMD and LBP, stratified by PIR.

Supplementary Image S9 The association between BMD and LBP, stratified by race.

Supplementary Image S10 The association between BMD and LBP, stratified by smoking status.
