# Supplementary figures and images for: Association between bone mineral density and lower back pain in the general United States population using the NHANES of 1999–2004
Source: Front Surg. 2025 Apr 10;12:1535614. doi: 10.3389/fsurg.2025.1535614 (PMC12035731; doi:10.3389/fsurg.2025.1535614)

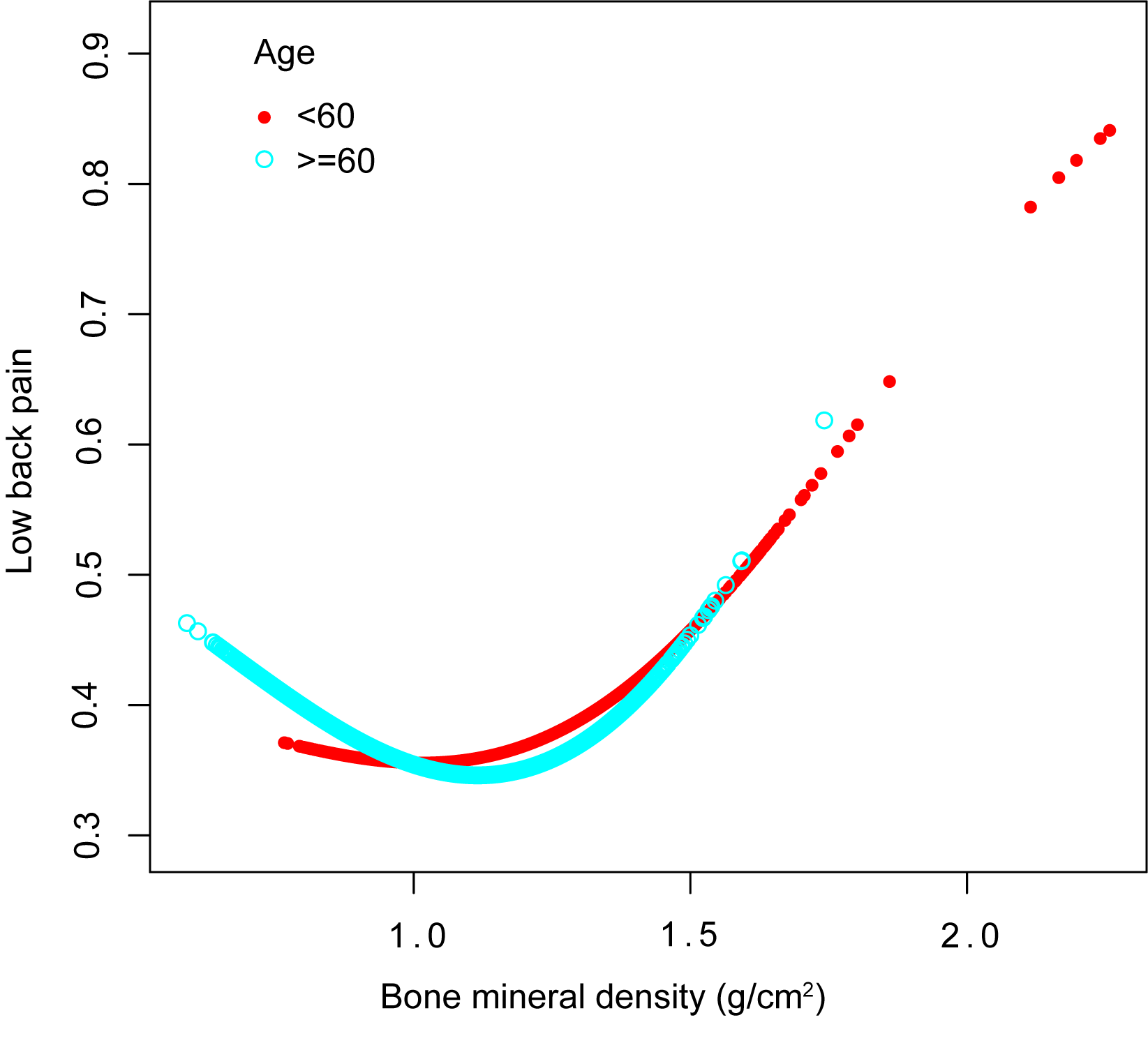

Supplement: Supplementary Image S1 — The association between BMD and LBP, stratified by age. [file Image1.tif]

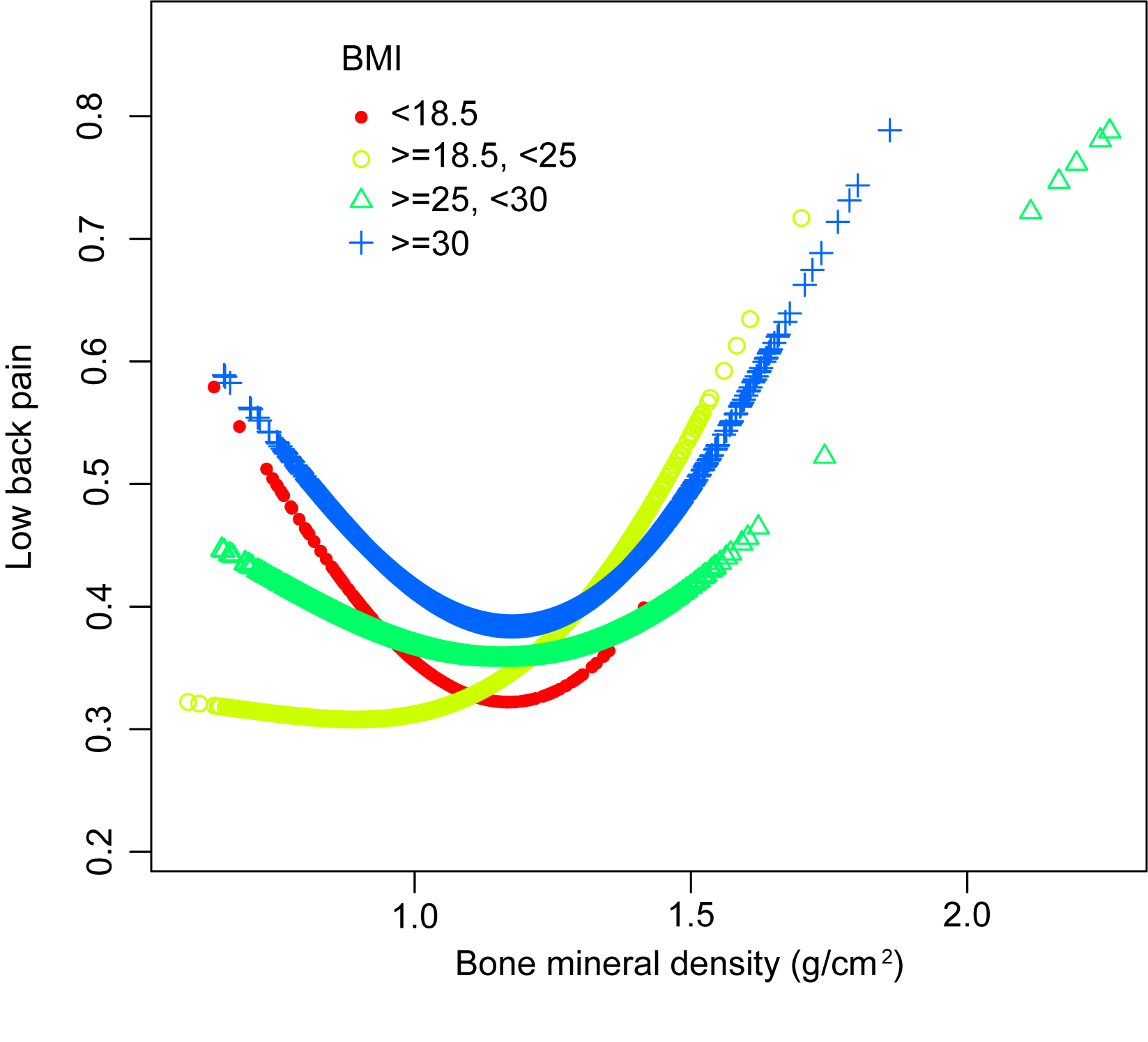

Supplement: Supplementary Image S2 — The association between BMD and LBP, stratified by BMI. [file Image2.tif]

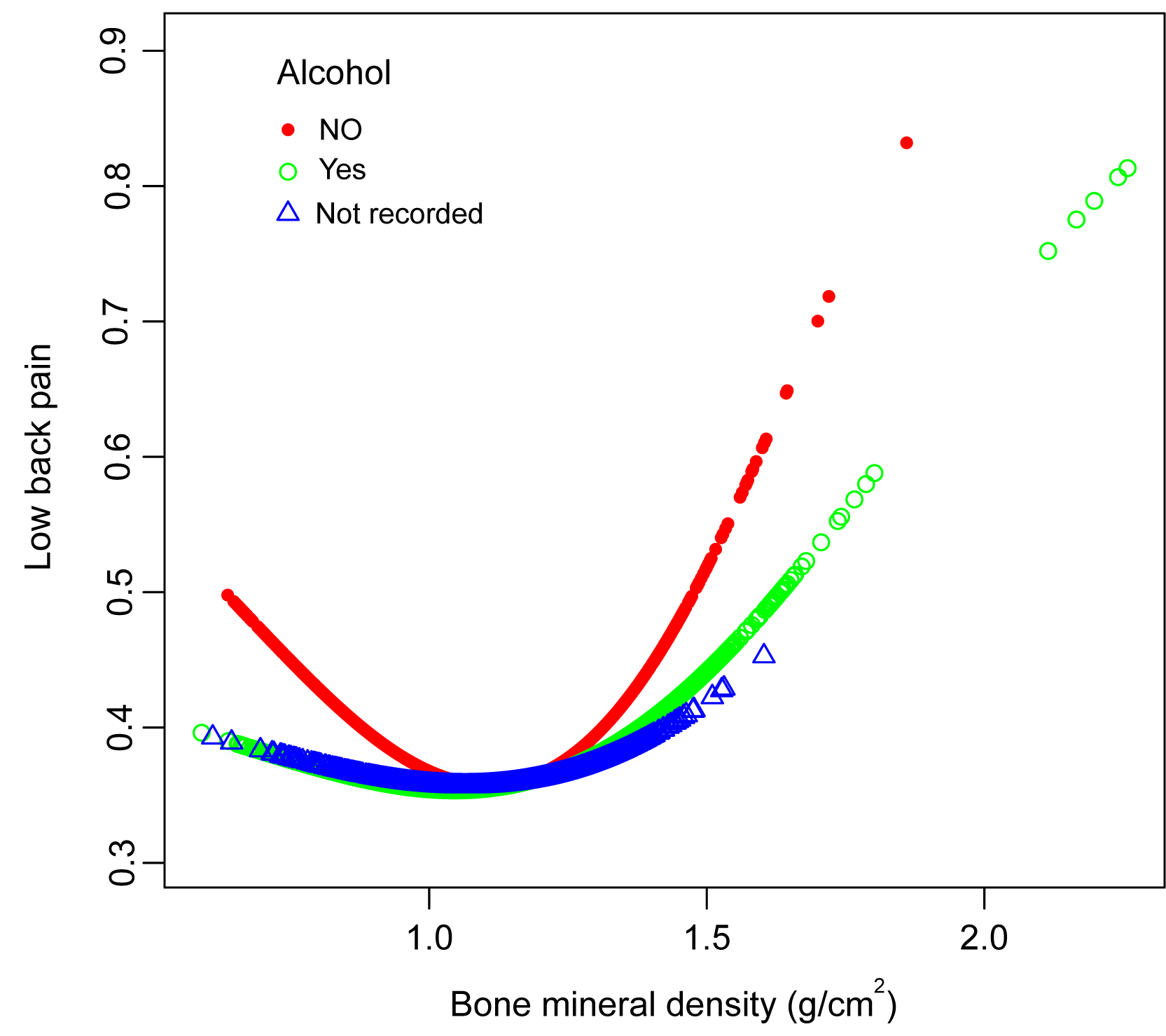

Supplement: Supplementary Image S3 — The association between BMD and LBP, stratified by alcohol. [file Image3.tif]

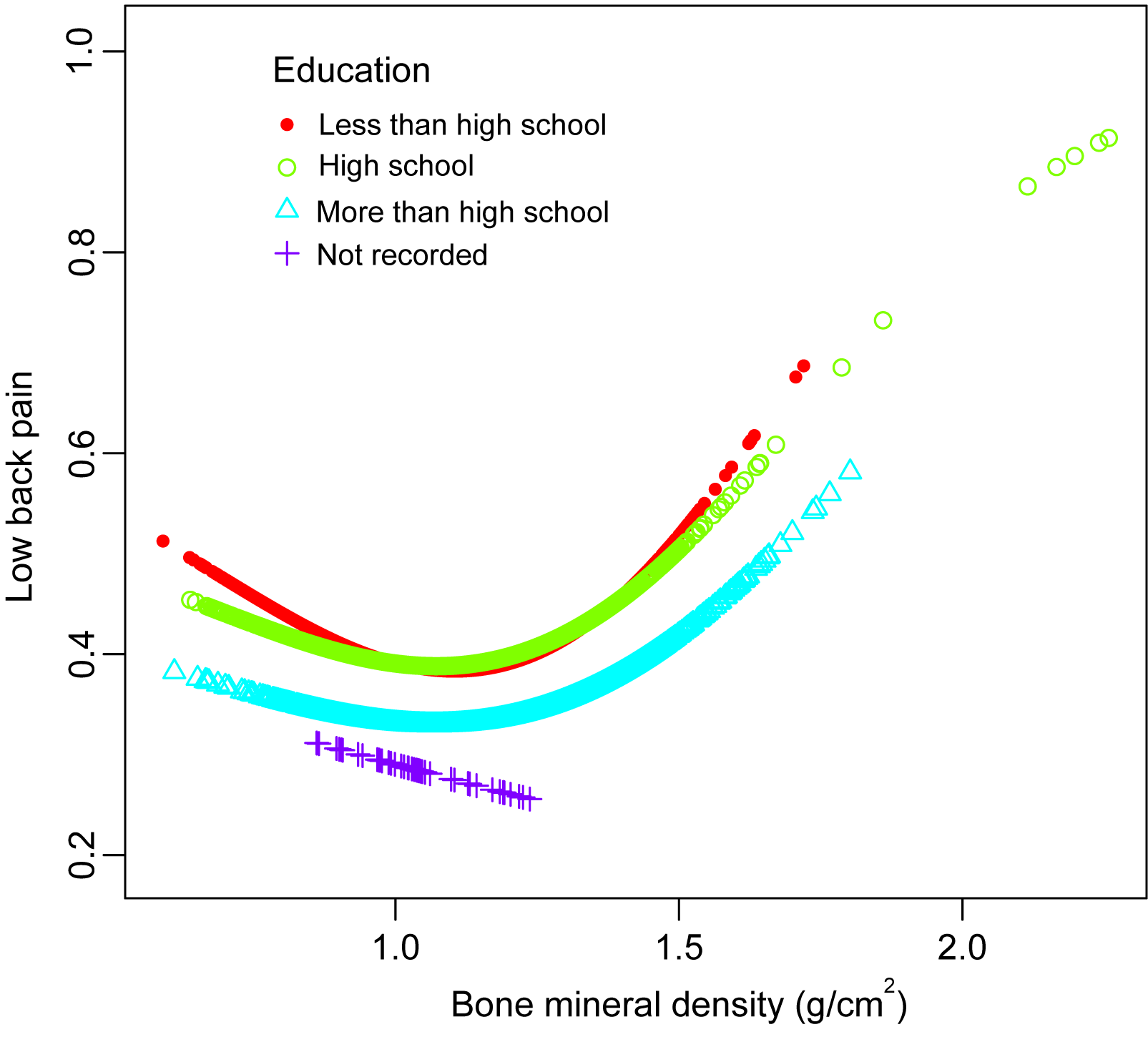

Supplement: Supplementary Image S4 — The association between BMD and LBP, stratified by education. [file Image4.tif]

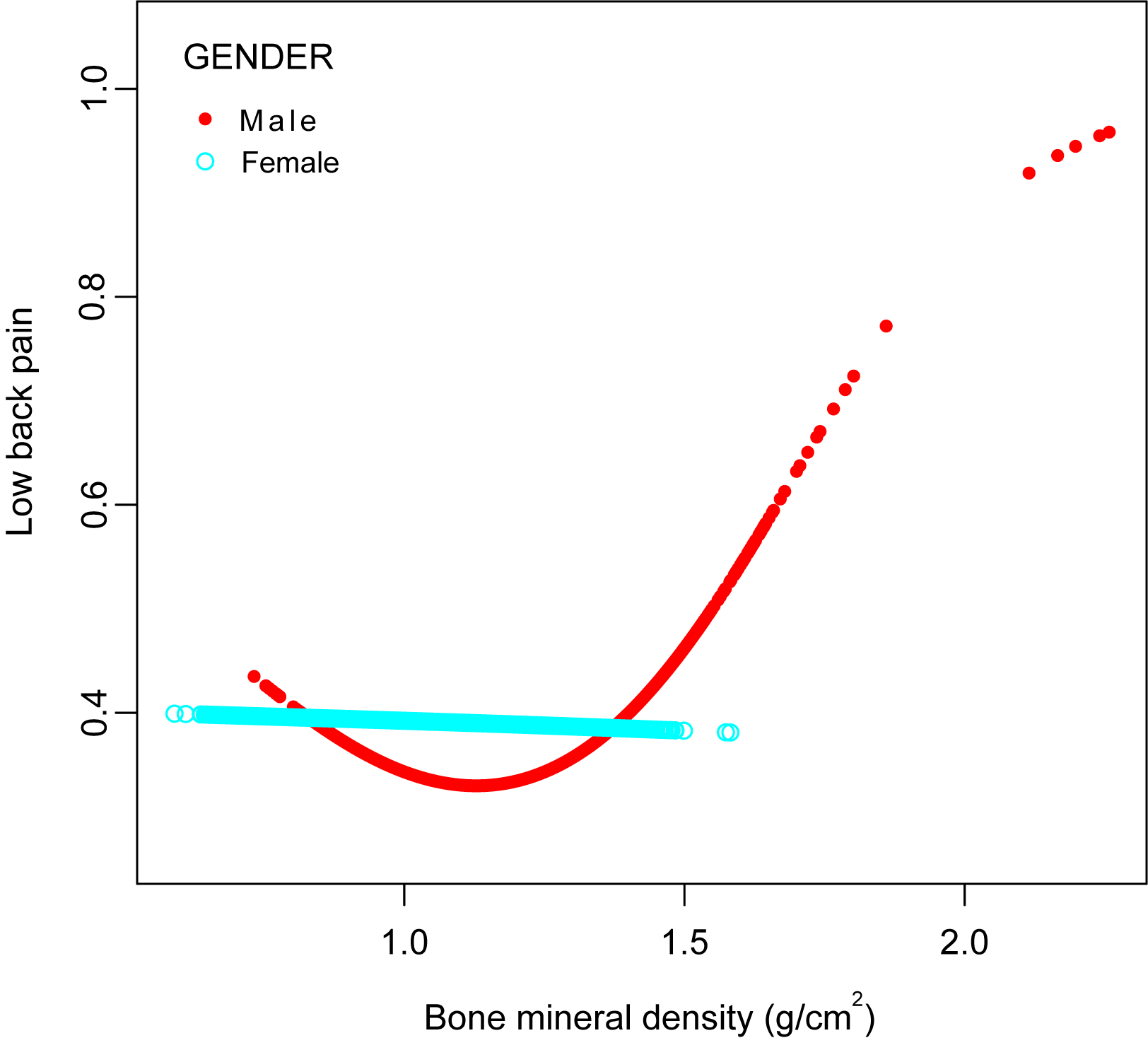

Supplement: Supplementary Image S5 — The association between BMD and LBP, stratified by Hyperlipidemia. [file Image5.tif]

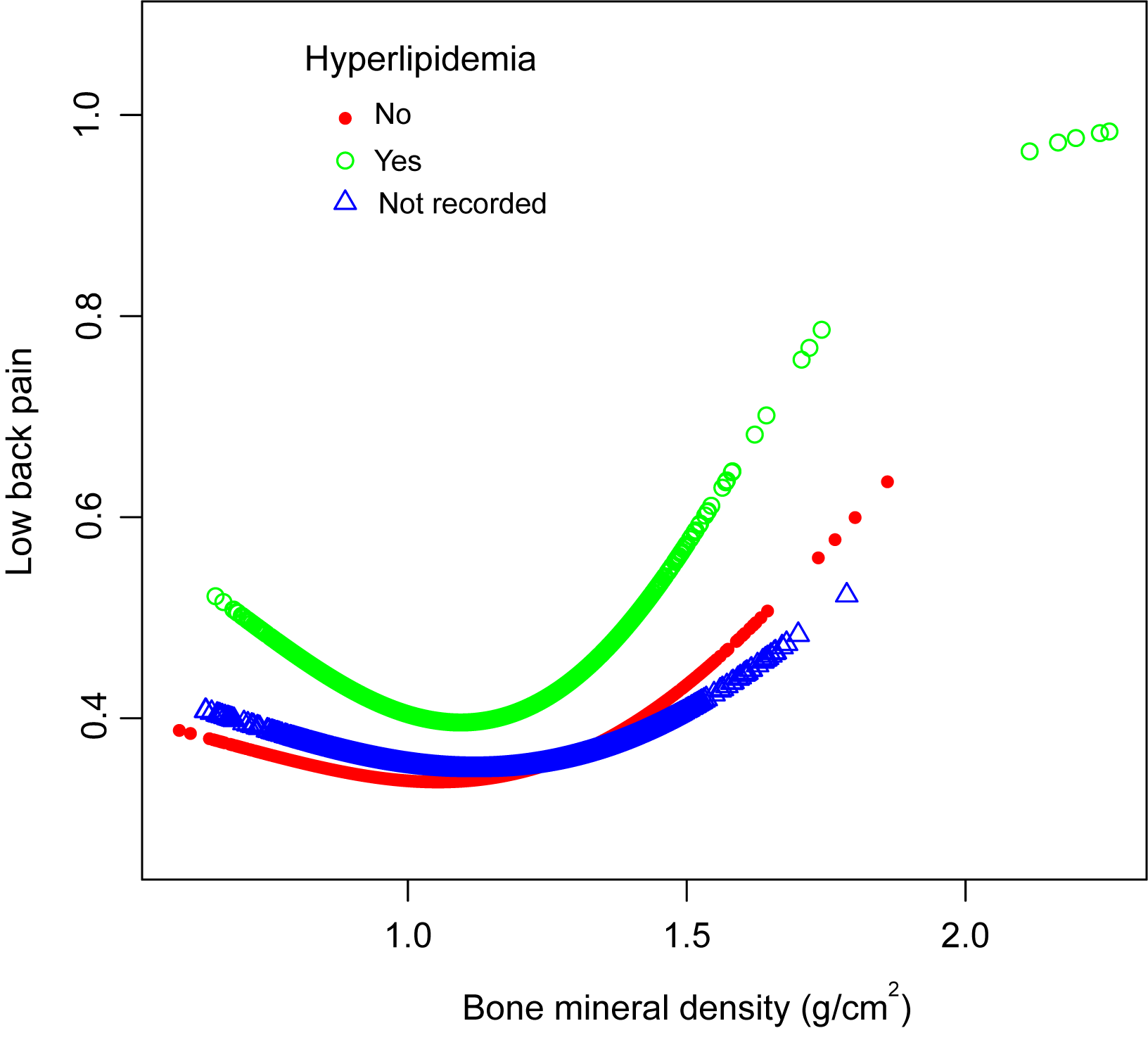

Supplement: Supplementary Image S6 — The association between BMD and LBP, stratified by Hyperlipidemia. [file Image6.tif]

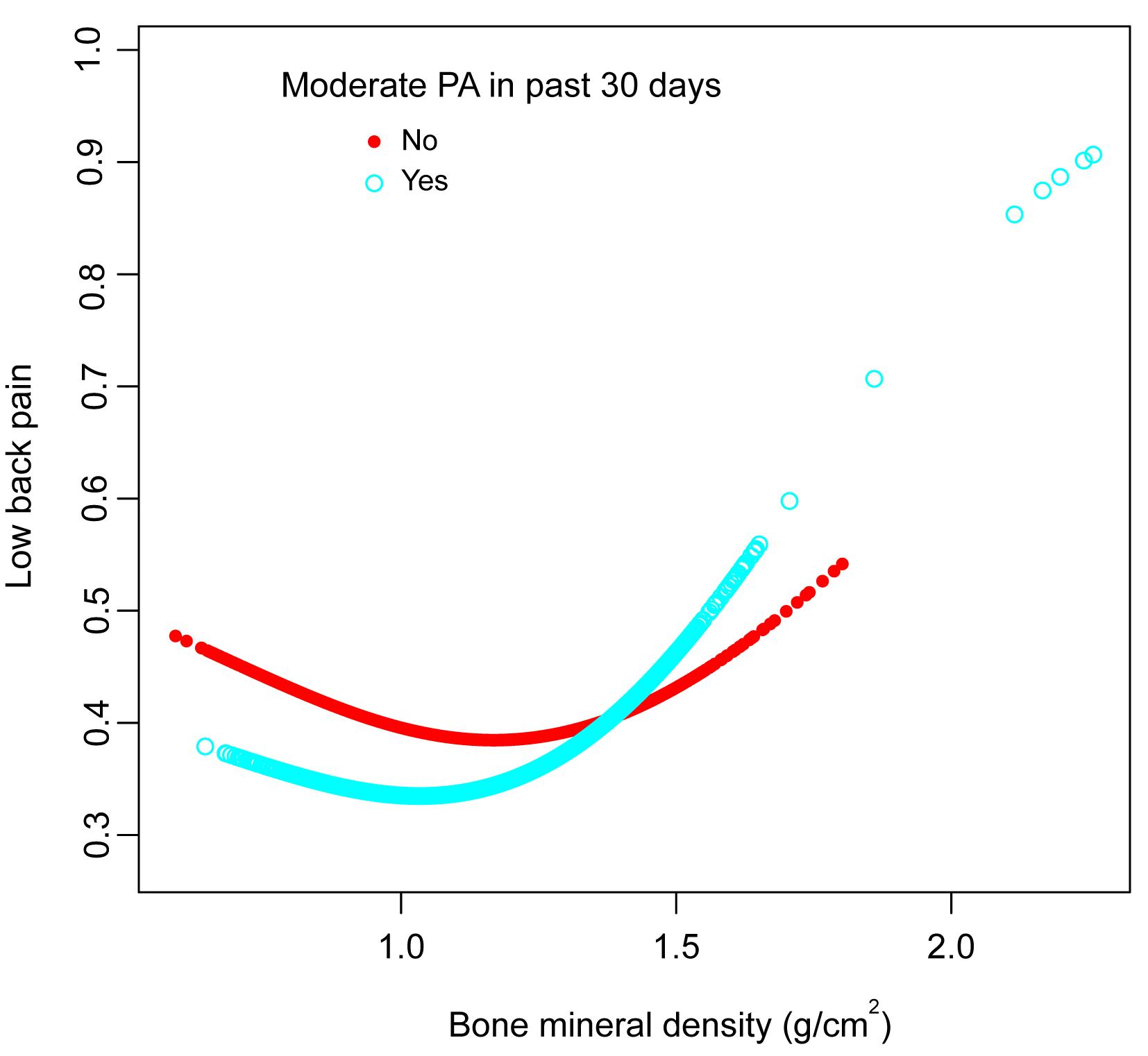

Supplement: Supplementary Image S7 — The association between BMD and LBP, stratified by moderate physical activity. [file Image7.tif]

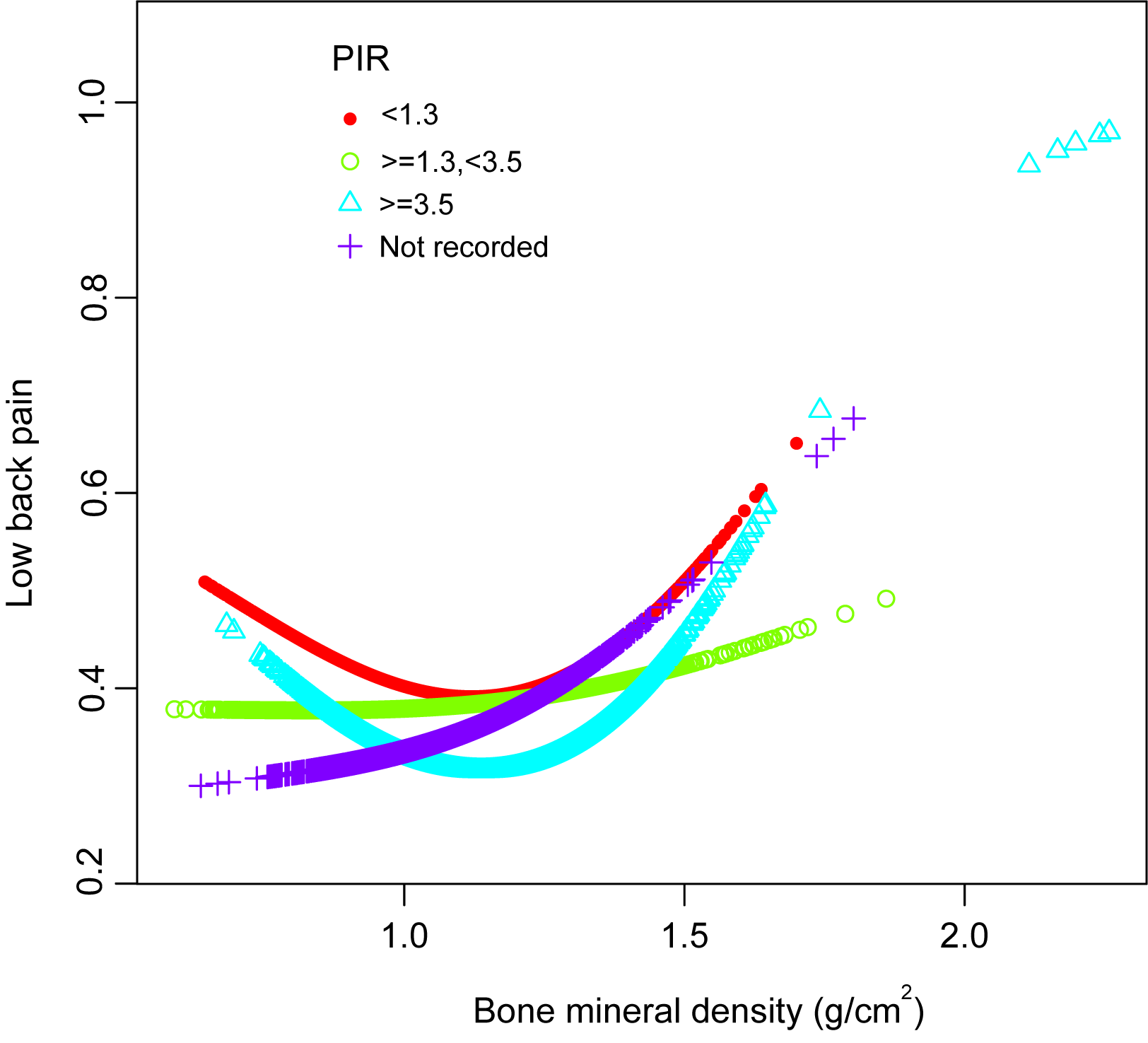

Supplement: Supplementary Image S8 — The association between BMD and LBP, stratified by PIR. [file Image8.tif]

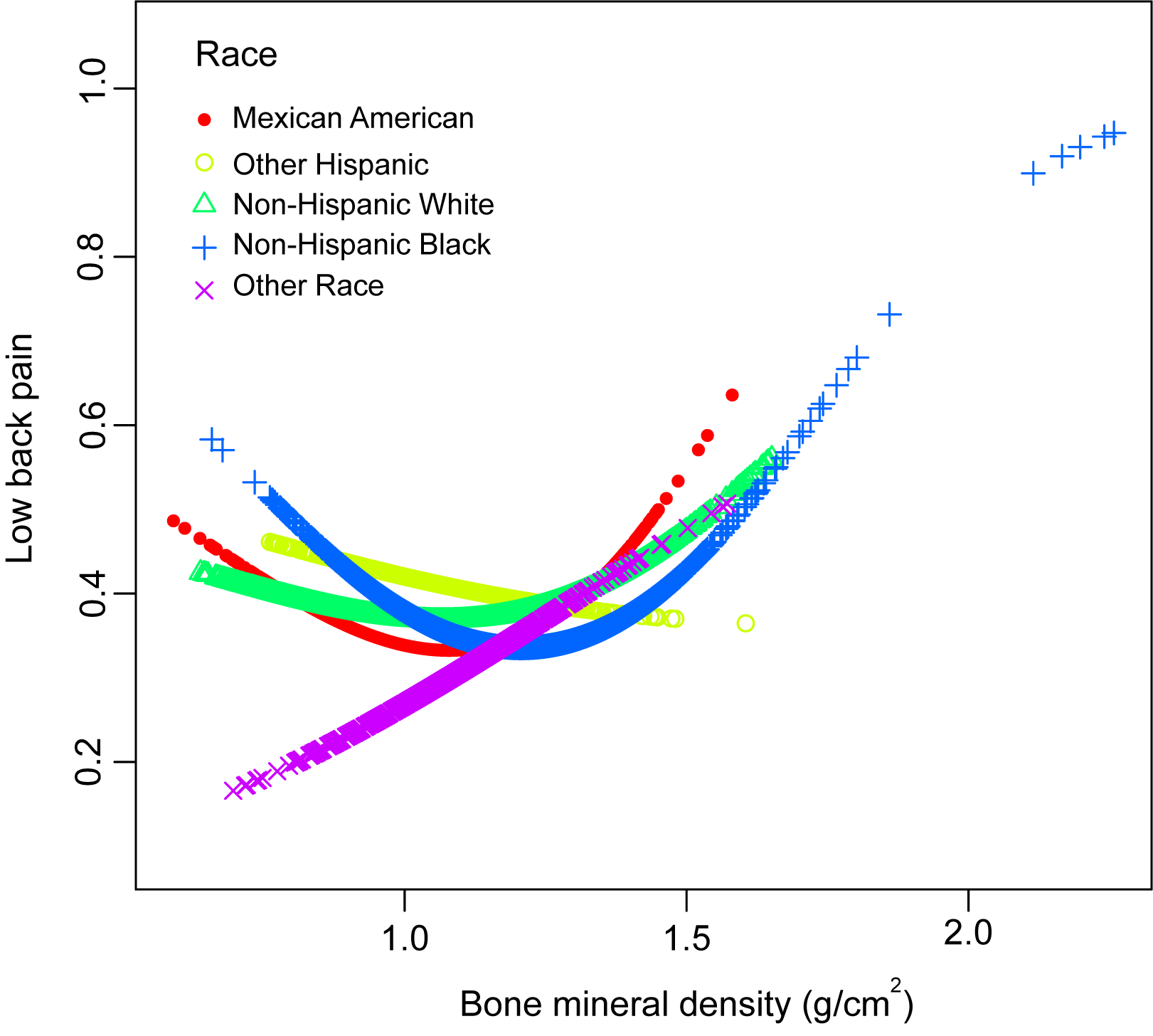

Supplement: Supplementary Image S9 — The association between BMD and LBP, stratified by race. [file Image9.tif]

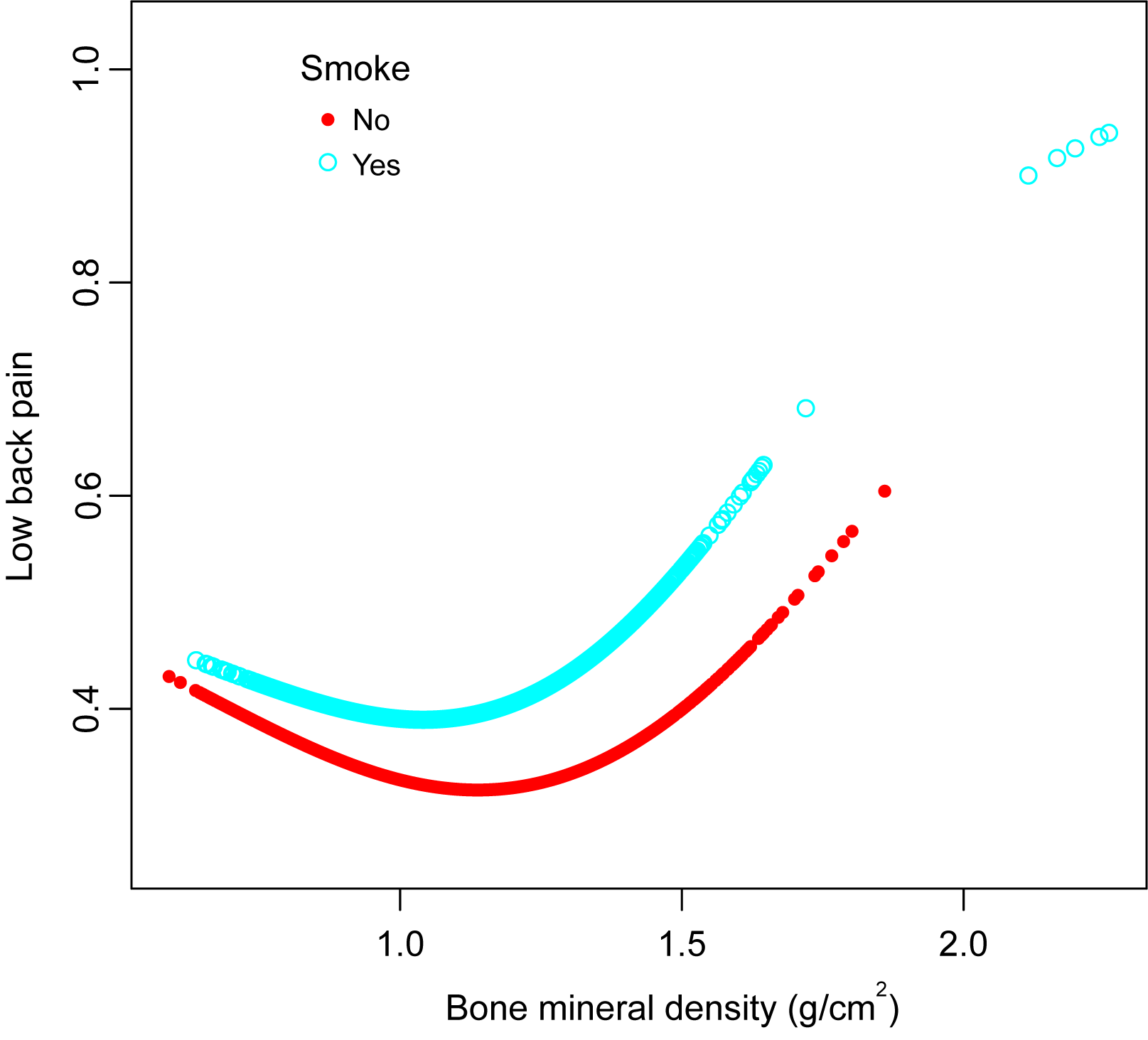

Supplement: Supplementary Image S10 — The association between BMD and LBP, stratified by smoking status. [file Image10.tif]
